# Phylogeny Predicts Future Habitat Shifts Due to Climate Change

**DOI:** 10.1371/journal.pone.0098907

**Published:** 2014-06-03

**Authors:** Matjaž Kuntner, Magdalena Năpăruş, Daiqin Li, Jonathan A. Coddington

**Affiliations:** 1 Institute of Biology, Scientific Research Centre, Slovenian Academy of Sciences and Arts, Ljubljana, Slovenia; 2 Centre for Behavioural Ecology and Evolution, College of Life Sciences, Hubei University, Wuhan, Hubei, China; 3 National Museum of Natural History, Smithsonian Institution, Washington, D. C., United States of America; 4 Centre of Landscape–Territory–Information Systems - CeLTIS, University of Bucharest, Bucharest, Romania; 5 Tular Cave Laboratory, Kranj, Slovenia; 6 Department of Biological Sciences, National University of Singapore, Singapore, Singapore; Pennsylvania State University, United States of America

## Abstract

**Background:**

Taxa may respond differently to climatic changes, depending on phylogenetic or ecological effects, but studies that discern among these alternatives are scarce. Here, we use two species pairs from globally distributed spider clades, each pair representing two lifestyles (generalist, specialist) to test the relative importance of phylogeny versus ecology in predicted responses to climate change.

**Methodology:**

We used a recent phylogenetic hypothesis for nephilid spiders to select four species from two genera (*Nephilingis* and *Nephilengys*) that match the above criteria, are fully allopatric but combined occupy all subtropical-tropical regions. Based on their records, we modeled each species niche spaces and predicted their ecological shifts 20, 40, 60, and 80 years into the future using customized GIS tools and projected climatic changes.

**Conclusions:**

Phylogeny better predicts the species current ecological preferences than do lifestyles. By 2080 all species face dramatic reductions in suitable habitat (54.8–77.1%) and adapt by moving towards higher altitudes and latitudes, although at different tempos. Phylogeny and life style explain simulated habitat shifts in altitude, but phylogeny is the sole best predictor of latitudinal shifts. Models incorporating phylogenetic relatedness are an important additional tool to predict accurately biotic responses to global change.

## Introduction

Biotic change due to global warming is increasing dramatically. Although climatic changes are increasingly well understood and future predictions are improving, it is much more difficult to predict biotic responses to climate change [1], [2]. Models predict global climates to become warmer in the following decades (Fig. S1), and precipitation patterns will change accordingly, but how organisms will respond to such changes is less clear. Naturally, different life histories and even clades of organisms may respond in various ways [3]. For example, they may respond with a shift in time, e.g. changing their phenology [2], [4], or a shift in space, such as moving to different habitats, latitudes, or altitudes [2], [5], [6], [7], insofar as habitats are available [8]. Crucial species interactions may be broken by both types of shifts [9]. Tropical species, in particular, may be severely affected [4], [10]. Actual adaptive evolutionary change within a few decades may be possible for some organisms with extraordinary genotypic and phenotypic plasticity [11], [12] but is unlikely for most as it would require over 10,000 times faster rates of adaptive change than those estimated [13]. Finally, the species unable to cope with anthropogenic global changes, and those whose dispersal ability is low, are likely to become extinct either globally or locally [1], [3], [14].

Communities, ecosystems and biomes are also likely to be forced to shift in space [2], become increasingly destabilized due to biotic loss [15], or be subject to thermophilization [16] and invasion [17]. In addition, food web and trophic interactions may be altered [18]. While biomes, notably tropical and subtropical forests, may expand in range due to increased rainfall and atmospheric CO_2_ levels [19], such trends are expected to be countered by human habitat degradation, probably resulting in net habitat loss [20]. In some cases, temperate assemblages may increase in species richness or exhibit a higher species turnover [21]. However, at the global level with all factors combined, most biomes and communities are predicted to deteriorate, and thus may simply shrink or disappear. Their space then may be taken over by exotic and invasive species and communities, whose success can sometimes be directly attributed to global change [17].

Terrestrial organisms may be most prone to global climate changes (but see freshwater and marine reviews [22], [23]), yet surprisingly few studies have modeled the future of closely related terrestrial lineages—clades—in order to discern among phylogenetic and ecological effects while studies modelling single species continue to abound [24], [25]. As an example of clade predictions, Diamond et al. [26] examined models for thermal tolerance in ants based on current and future climates and found that tropical ants had lower tolerances to warming. Tropical ant faunas, thus, are more susceptible to global warming, and that is precisely where the most diversity lies. However, is this necessarily true for all tropical ants? One would expect that phylogeny may affect biotic responses to climate change at least as much as ecology or natural history [27]. Fine grained studies discerning among these factors are currently lacking [3].

**Figure 1:**
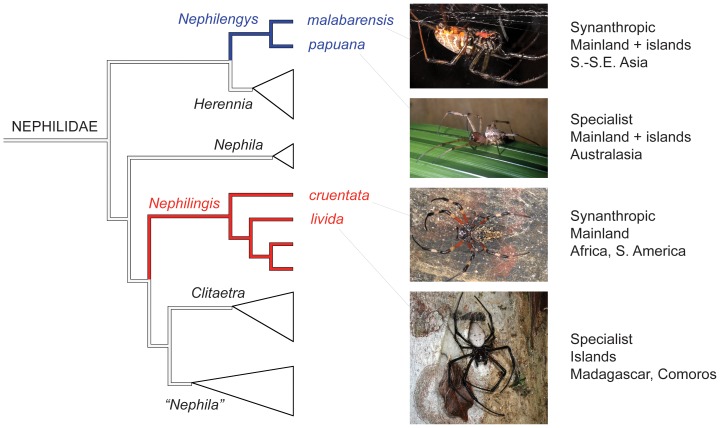
Two nephilid species pairs, their phylogeny, and basic ecology. One species from each clade is synanthropic and the other one a habitat specialist. This sample tests the relative importance of phylogeny versus life history on species responses to climatic changes. The phylogenetic hypothesis builds on a nephilid species level study that used 4kB of nucleotide data in addition to morphology, and an array of analytical approaches and sensitivity analyses [28].

**Figure 2:**
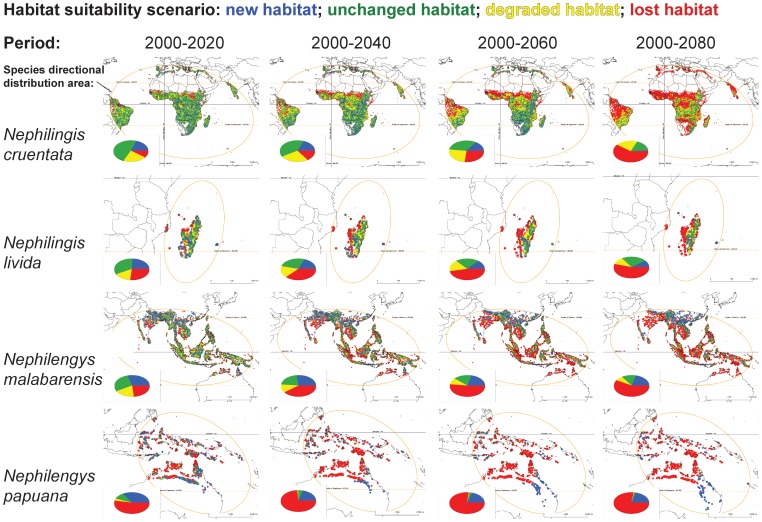
Predicted habitat suitability changes of two *Nephilingis* and two *Nephilengys* species over increasing time ranges. The predictions are based on the IPCC scenario A1B for temperature and precipitation (see Figs S1–S2) and are modeled for the time ranges 2000 vs. 2020, 2040, 2060, and 2080. Future habitat is classified as new, unchanged, degraded or lost.

To test the relative importance of phylogeny versus ecology in predicted biotic responses to climate change, we selected two clades within which species have occupied two distinct ecological niches. The spider genera *Nephilengys and Nephilingis* [28] are large terrestrial invertebrate predators. The Asian *Nephilengys malabarensis* is synanthropic and hence relatively generalized, and the Australasian *Ny. papuana* is a forest species, thus relatively specialized [29]. Likewise the African-South American *Nephilingis cruentata* is synanthropic, whereas the Madagascan-Comoroan *Ni. livida* is a forest species (Fig. 1) [29], [30], [31]. The four species are fully allopatric, but combined they occupy all subtropical-tropical regions [29]. Ecologically the two specialists, *Ni. livida* and *Ny. papuana* should be more prone to habitat loss than the generalists *Ny. malabarensis* and *Ni. cruentata*. This prediction derives from the assertions that species with greater ecological generalization should be more likely to shift to higher elevation and latitude [3] and that niche breath positively correlates with species geographic ranges [32]. Alternatively, phylogenetic relatedness may better explain response trends. We first used predicted temperature and precipitation changes (Fig. S1) and a GIS simulation tool to model habitat degradation, gain, and loss in the four species (Fig. 2, Fig. S2) at 20 year intervals from 2000–2080. We then investigated the extent to which phylogeny versus ecology explained habitat changes. A priori predictions are difficult: if commonly derived species undergo life history adaptation to reduce competition, then ecology may be a better predictor. If not, phylogeny may be used to predict habitat shifts due to climate change.

## Materials and Methods

### Modeling current habitat suitability

We used all available locality data for the four species [29]: *Ni. cruentata* (specimen records N = 436), *Ni. livida* (N = 138), *Ny. malabarensis* (N = 138) and *Ny. papuana* (N = 39). Since our previous study phylogenetic evidence has emerged placing *Ni. cruentata* and *Ni. livida* in the genus *Nephilingis*, which is phylogenetically distant from *Nephilengys* containing *N. malabarensis* and *Ny. papuana* [28]. This is currently the best available phylogenetic hypothesis of nephilid species relationships, having utilized 4 kB of nucleotide data in addition to morphology, and an array of analytical approaches and sensitivity analyses [28]. That the previously congeneric species in fact belong to distinct clades, which nevertheless contain ecologically dissimilar species (one synanthropic and one habitat specialist within each genus), adds power to our testing of phylogenetic versus life history effects on habitat suitability. Our previous model selected best fit pairs of ecological parameters for each species current habitat suitability assessment based on the outcomes of backward linear regression: annual mean temperature and elevation was used for the *Ni. cruentata* model, global land cover and elevation for the *Ni. livida* model, and global land cover and annual mean precipitation for both *Ny. malabarensis* and *Ny. papuana* models [29]. Here, we used the same pairs of parameters for all four species models, which are indicative of global climate change: annual mean temperature (TMA) and annual mean precipitation (PMA). Based on these two parameters, we modeled the starting point for the year 2000, taken as each species' current habitat suitability.

### Modeling future habitat suitability

We downloaded the raster data for TMA and PMA for the years 2020 to 2080 from the Downscaled Global Circulation Models data portal (http://www.ccafs-climate.org), and the current distribution (1950–2000) of climates from the WorldClim Database [33]. The raster layers are based on the IPCC Special Report on Emissions Scenarios (SRES) [34], where several scenario families of predicted climate change were proposed. The scenario A1 builds on the assumption of maximum energy requirements, and the sub-choices are based on emissions expected from the use of different fuel sources: fossil intensive (A1F1), technologically developed non-fossil sources (A1T) and a balance across sources (A1B). Among the worst case scenario (A1) we chose the balanced use of fuel sources (A1B) as the basis for our models. The remaining three scenarios build on minimum energy requirements and emissions (B1), on high energy requirements (A1F1) and on lower energy requirements; however, as the scenarios A2 and B2 only predict a limited number of years (2020, 2050 and 2080), and the A1 scenario predicts each decade, our choice of scenario (A1B) seemed justified. For global modeling use, IPCC recommends the use of A1 and B1 [35], among which we chose to model a more pessimistic version due to the availability of predicted climate change (for B1 no associated datasets exist). The A1B scenario uses 24 Global Circulation Models [36]. All rasters have a spatial resolution of 2.5 arc-minutes, using a WGS84 datum.

The A1 group of scenarios predicts a global average surface warming until 2100 from 1.4 to 6.4°C [35]. Fig. S1 shows projected global changes in TMA and PMA for the period covered by our models (current  = 2000; projected 2020, 2040, 2060, 2080). Based on these, we modeled habitat suitability of each of the four species following our GIS modeling methodology [29]. These maps define each species' directional distribution as its potential target area and thus potential dispersal range [29], and within it, we used the frequency distribution values for TMA and PMA for current specimen records [29]. We here explore the changes for each species in three habitat categories: high, moderate and low habitat suitability (Fig. S2). Several data sources exist that model global changes according to the A1B scenario. We choose the data from the Canadian Centre for Climate Modelling and Analysis - CGCM3.1 (T47), 2005 [37]. This data model version is the basis for the suite of model simulations in the IPCC Fourth Assessment Report [35]. T47 version of CGCM3.1 data model has a surface grid whose spatial resolution is roughly 3.75 degrees latitude/longitude and 31 levels vertically, and an ocean grid resolution of roughly 1.85 degrees, with 29 levels vertically [38], [39].

### Assessing species habitat loss

To assess the degrees of projected habitat losses or gains due to climatic changes, we compared the differences in predicted habitat suitability categories for the following time periods: 2000–2020, 2000–2040, 2000–2060, and 2000–2080. We customized our previous GIS model [29] with additional tools in ArcGIS [40]: i) First, we reclassified each year's habitat suitability raster data where no habitat suitability was shown from ‘NoData’ to ‘99’ using the tool ‘Reclassify’; those values that showed a habitat suitability value were left as they were (high = 3; moderate = 2; low = 1); ii) We then compared using the ‘Combine’ tool the habitat category changes between the year 2000 and other time periods and reclassified the resulting combinations into the following nominal values: 1 = new habitat (indicating projected habitat suitability gain); 2 = unchanged habitat (indicating no change in habitat suitability); 3 = degraded habitat (indicating a loss in habitat suitability value, but not below 1) and 4 =  lost habitat (indicating a complete loss of projected habitat suitability, i.e. resulting in a change to 99).

### Statistical analyses

Since the TMA and PMA data were not normally distributed, we employed standard non-parametric statistics to test the effects of phylogenetic relatedness (*Nephilingis* vs. *Nephilengys*) and life history (synanthropic vs. habitat specialist) on current species ecological preferences. To determine whether phylogenetic or ecological factors are good predictors of the simulated habitat shifts, we first devised a randomized dataset of 10,000 simulated georeferenced records per period per species (totaling 200,000 records; Table S1), calculated latitudinal and altitudinal shifts and coded them as categorical data (i.e., shift or not) based on the shifted values. We performed generalized linear model (GLM) using ordinal logistical model twice, once for latitudinal shifts and once for altitudinal shifts, with phylogeny, life style, period (2020, 2040, 2060 and 2080) and habitat suitability (high = 3; moderate = 2; low = 1) as predictors. Both period and habitat suitability showed the same effects on shifts in the initial GLM tests, thus being excluded from the final models, which tested the effects of only two factors (phylogeny and life style) and the 2-way interaction on the categorical latitude and altitude shifts. The model was fitted using multinominal distribution with cumulative logit link error. Akaike information criterion (AIC: the smallest is the best) was used to select the best model. All statistical analyses were done using IBM SPSS Statistics 21 (IBM Corporation, USA). Reported *p*-values are two-tailed tests, with α = 0.05.

## Results

**Figure 3:**
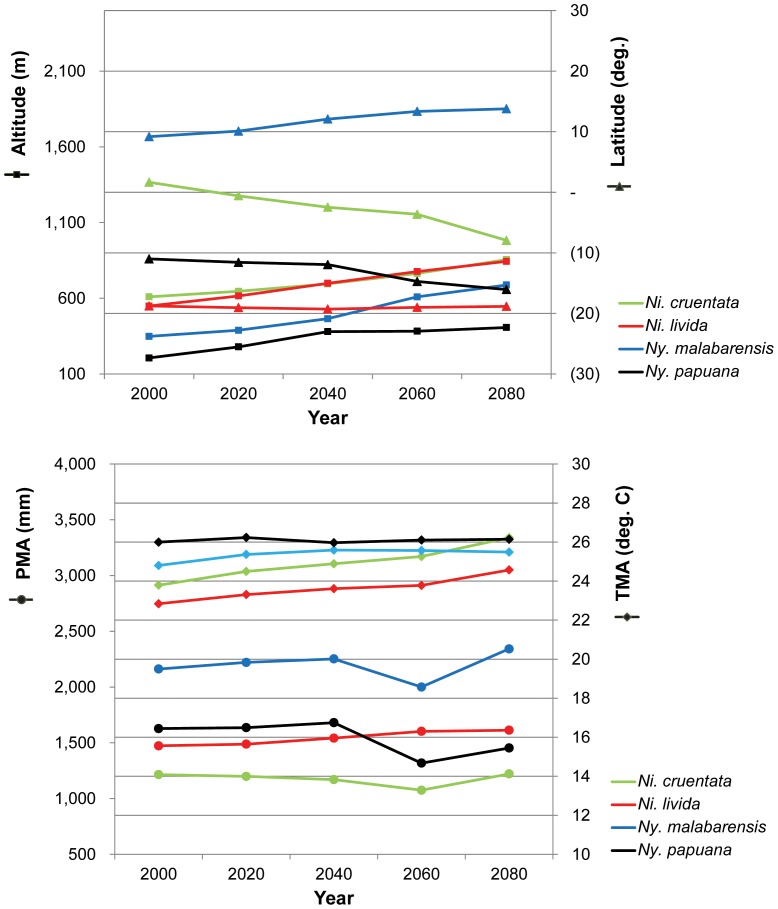
Changes for all species in altitude and latitude (above), and temperature and precipitation (below). Species maintain their characteristic climatic conditions by shifting towards higher altitudes and latitudes. Geographical and ecological averages for the modeled suitable habitats combined all categories (low, medium and high suitability). NI = *Nephilingis*, NG = *Nephilengys*, PMA = mean annual precipitation, TMA =  mean annual temperature.

The global habitat suitability models for 2000, 2020, 2040, 2060 and 2080 predict overall shrinking or disappearance of suitable habitat for all four species (Fig. S2), although minor amounts of newly appropriate habitat emerge over time. Categorizing habitat change as unchanged, degraded, lost, or new, the major trend across all time ranges is dramatic increase in either lost or degraded habitat for all species (Fig. 2; Table 1). Losses and gains can take different tempos. *Ni. cruentata* shows a dramatic loss by 2080, but in *Ny. papuana* the most dramatic loss occurs by 2040. Others are intermediate. In no case does new habitat tend to increase (Table 1). By 2080, habitat loss ranges from 54.8% (*Ni. livida*) to 77.1% (*Ny. papuana*). Even the widespread, generalist species *Ni. cruentata* and *Ny. malabarensis* lose 60.6% and 58.6% of their habitat. According to the model, all four species roughly maintain their known temperature and precipitation preferences (Fig. 3). However in order to do so, all species shift towards higher altitudes and latitudes (except *Ni. livida*, whose island habitats are obviously fixed) (Fig. 3).

**Table 1:** Predicted percentages of future habitat change per species relative to the year 2000.

Species	Habitat categories	2020	2040	2060	2080
***Nephilingis cruentata***	new	19.9%	19.2%	16.1%	1.2%
	unchanged	48.5%	40.0%	31.7%	17.8%
	degraded	20.9%	25.1%	25.6%	20.4%
	lost	10.7%	15.7%	26.6%	60.6%
***Nephilingis livida***	new	24.5%	19.1%	14.2%	10.6%
	unchanged	33.9%	26.6%	21.6%	23.3%
	degraded	15.2%	17.8%	17.7%	11.3%
	lost	26.4%	36.5%	46.5%	54.8%
***Nephilengys malabarensis***	new	29.3%	26.8%	21.5%	21.0%
	unchanged	29.4%	21.6%	16.8%	13.2%
	degraded	17.8%	12.5%	9.7%	7.2%
	lost	23.5%	39.1%	52.0%	58.6%
***Nephilengys papuana***	new	33.3%	20.8%	20.2%	20.9%
	unchanged	10.5%	4.2%	2.5%	1.5%
	degraded	3.2%	1.0%	0.7%	0.5%
	lost	53.0%	74.0%	76.6%	77.1%

Phylogeny significantly explains variation in current temperature preferences (Mann-Whitney U = 24,965, *df* = 1, *p*<0.0001). Both phylogeny and lifestyle significantly explain current precipitation preferences, but phylogeny much more strongly than lifestyle (Mann-Whitney U = 25,803, *df* = 1, *p*<0.0001 and U = 13,503, *df* = 1, *p*<0.016).

GLM analyses on simulated data show that phylogeny predicts latitudinal shifts better than life style does (Goodness of fit: AIC = 116.261; Omnibus test: *χ* ^2^ = 328.2, *df* = 3, *p*<0.0001; Table 2): *Nephilengys* is more likely to shift latitude than *Nephilingis*. Both phylogeny and life style are good predictors of the simulated altitudinal shifts (Goodness of fit: AIC = 107.247; Omnibus test: *χ* ^2^ = 48.242, *df* = 3, *p*<0.0001; Table 2): altitudinal shifts are more likely in *Nephilingis* than *Nephilengys* and in synanthropic species than in habitat specialists.

**Table 2:** Results from a generalized linear model (GLM) testing the effects of two explorative factors (phylogeny and lifestyle) on how spiders respond to climate conditions (based on the IPCC scenario A1B for temperature and precipitation) by shifting latitude or altitude.

Shift	Exploratory factors	Wald *χ* ^2^	*df*	*p*
Latitude	Phylogeny (genus-pair)	310.711	1	<0.0001
	Life style	3.65	1	0.056
	Phylogeny × Life style	13.730	1	<0.0001
Altitude	Phylogeny (genus-pair)	30.825	1	<0.0001
	Life style	17.350	1	<0.0001
	Phylogeny × Life style	0.082	1	0.775

The model that included two factors and 2-way interaction was fitted using multinominal distribution with cumulative logit link error.

GLM test for latitude shift: Goodness of fit: AIC = 116.261; Omnibus test: *χ* ^2^ = 328.2, *df* = 3, *p*<0.0001; GLM test for altitude shift: Goodness of fit: AIC = 107.247; Omnibus test: *χ* ^2^ = 48.242, *df* = 3, *p*<0.0001.

## Discussion

Models that forecast biodiversity patterns have been grouped into four categories [41]: 1) those considering individual species, 2) those grouping species by niche, 3) those based on general circulation or coupled ocean-atmosphere-biosphere theories, and 4) those based on species-area curves [41]. We argue that models using phylogenetic relatedness should also be considered because our results have shown that phylogeny can strongly predict climatic and habitat preference.

Studies considering individual species fail to account for species interactions and phylogenetic factors and are therefore of limited general use. In one case, the already limited range of the golden striped salamander decreased even more [42]; in another an invasive species, the Australian redback spider, increased its already substantial distribution [43]. Outside of a phylogenetic context, these patterns make no general predictions for evolutionarily or ecologically closely related organisms.

More taxonomically inclusive studies often predict changes in large clades of organisms, notably amphibians [44], [45] or ants [26], but again, fine-grained clade-based studies are too preliminary. For example, the ranges of European amphibians and reptiles are generally predicted to shrink by 2050 [44], but this study was based on two opposite, and equally unlikely assumptions of unlimited versus no dispersal. A study of plant, bird and mammal assemblages in Europe only found a weak relationship between phylogeny and climate change vulnerability [46]. However, Willis et al. [47] investigated the changes in phenology and abundance of a temperate flora over 150 years and found that different clades are quite differently affected by climate change.

Niche modelling is limited as it fails to account for differences in species natural histories [3]. Only recently have studies taken into account species trait variability in assessing predicted responses to climate change. Angert et al. [3] examined to what extent species' traits are predictive of expanding their ranges. Predicting ecologically general species with greater dispersal abilities to more easily shift to higher latitudes and altitudes in response to climatic changes, they did find the expected relationships in passeriform birds and odonates, but their models yielded low explanatory power.

Our study extended this logic on a phylogenetically fine-grained taxonomic sample, by testing the relative importance of phylogeny versus species traits in predicted biotic responses to climate change in two species pairs from globally distributed spider clades, each pair representing two lifestyles. Despite the prediction that the unrelated specialists (forest-dwelling species) would cope with projected global changes worse than the unrelated generalists (synanthropic species), our results project habitat shrinkage patterns that may better be predicted by phylogeny than life style.

Our modeling approach assumes genotypic and phenotypic species homogeneity, perhaps an unwarranted assumption considering wide geographical ranges these species occupy. In reality, even widespread generalists show considerable genetic and phenotypic adaptations, spiders being no exception [48]. The predicted habitat shifts therefore may not be equally likely throughout the entire range of these taxa, and it may be possible that levels of adaptive change could take place at short temporal scales such as these presented in our study. The modes of habitat loss and extinction may depend on many additional factors, such as geography or basic natural history that are too complex to model accurately [3]. Phylogeny—which does broadly predict many life history traits—can simplify the prediction of biotic responses to future climate change. For accurate modelling, of course, many other factors are important as well, such as species interactions [49], [50], persistence abilities [51], population dynamics and changes in genetic diversity [52]. However, more work assessing phylogeny versus ecology and life history as explanations for responses to climate change is needed.

## Supporting Information

Figure S1 **Predicted global changes in temperature and precipitation based on the IPCC scenario A1B.** These predicted changes were used as bases for modeling species distribution 2000–2020, 2000–2040, 2000–2060 and 2000–2080.
(TIF)

Figure S2 **Models predicting future habitat suitability for two** ***Nephilingis*** **and two** ***Nephilengys*** **species.** The models for the time periods 2000, 2020, 2040, 2060 and 2080 are based on the IPCC scenario A1B for temperature and precipitation changes (see Fig. S1).
(TIF)

Table S1 **Simulated geo-records per period per species (data visualized in Fig. S1).**
(TXT)
