# Supplementary figures and images for: Phylogeny Predicts Future Habitat Shifts Due to Climate Change
Source: PLoS One. 2014 Jun 3;9(6):e98907. doi: 10.1371/journal.pone.0098907 (PMC4044009; doi:10.1371/journal.pone.0098907)

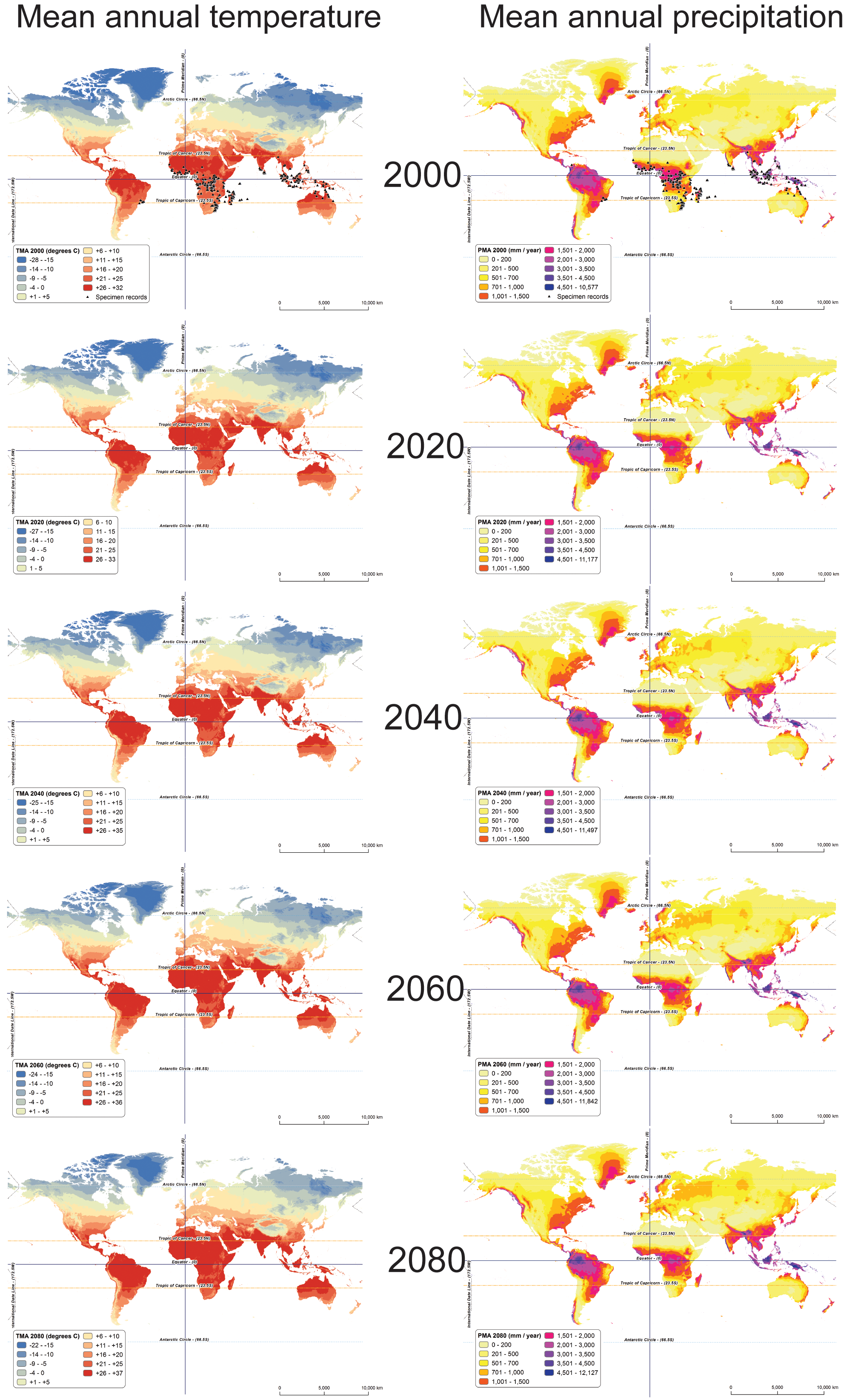

Supplement: Figure S1 — Predicted global changes in temperature and precipitation based on the IPCC scenario A1B. These predicted changes were used as bases for modeling species distribution 2000–2020, 2000–2040, 2000–2060 and 2000–2080. (TIF) [file pone.0098907.s001.tif]

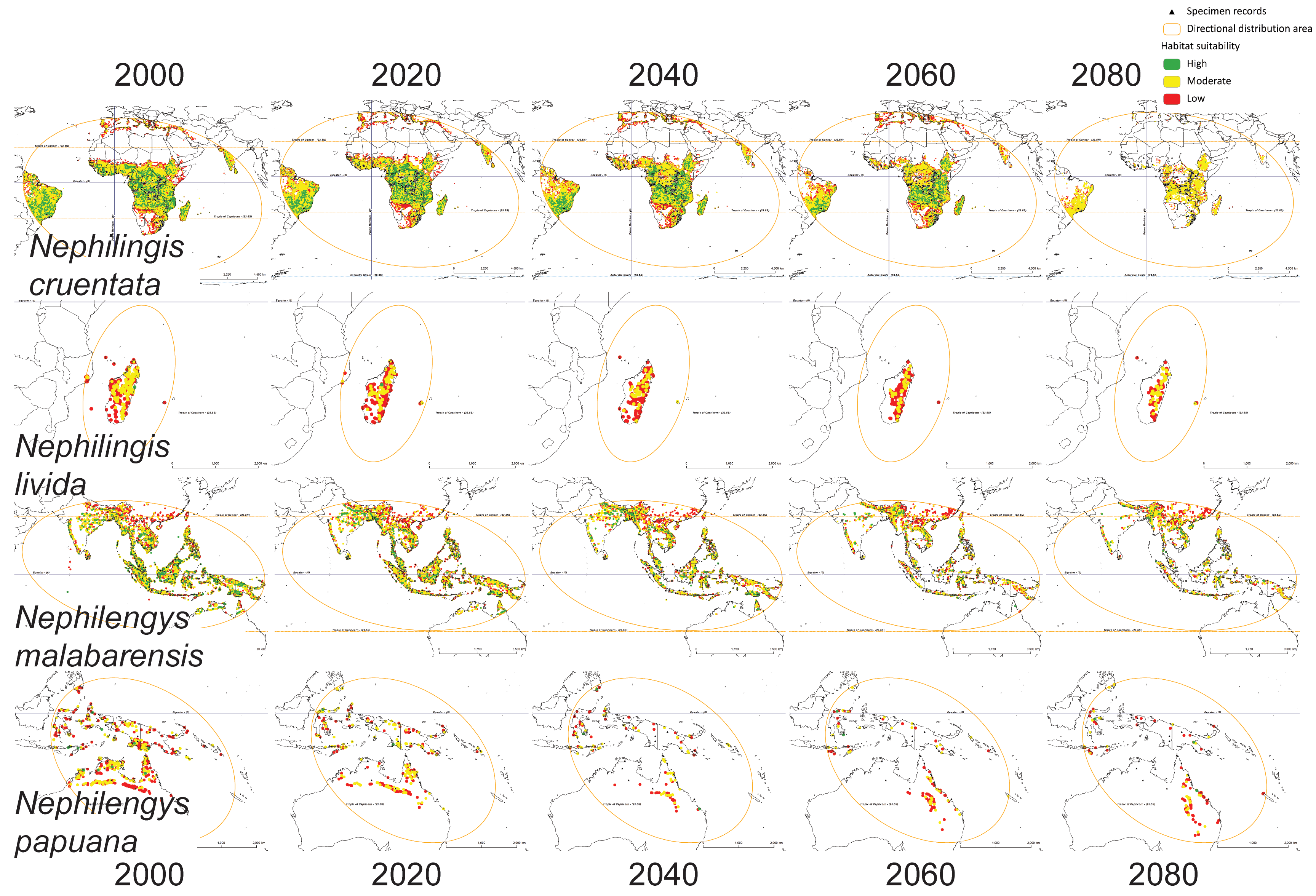

Supplement: Figure S2 — Models predicting future habitat suitability for two Nephilingis and two Nephilengys species. The models for the time periods 2000, 2020, 2040, 2060 and 2080 are based on the IPCC scenario A1B for temperature and precipitation changes (see Fig. S1). (TIF) [file pone.0098907.s002.tif]
